# lncRNA TINCR Regulates Proliferation and Invasion of Hepatocellular Carcinoma Cells by Regulating the miR-375/ATG7 Axis

**DOI:** 10.1155/2022/8132403

**Published:** 2022-09-16

**Authors:** Chengwu Tang, Hongbin Yu, Yinyuan Zheng, Wenming Feng, Jiasheng Shen

**Affiliations:** ^1^Department of General Surgery, First People's Hospital Affiliated to Huzhou Normal College, Huzhou, 313000 Zhejiang, China; ^2^Huzhou Key Laboratory of Translational Medicine, First People's Hospital Affiliated to Huzhou Normal College, Huzhou, 313000 Zhejiang, China; ^3^Department of Radiology, First People's Hospital Affiliated to Huzhou Normal College, Huzhou, 313000 Zhejiang, China

## Abstract

**Purpose:**

The aim of this study was to examine the role of the long noncoding RNA (lncRNA) terminal differentiation-induced noncoding RNA (TINCR) on the proliferation, apoptosis, and invasion of liver cancer cells and its mechanism.

**Methods:**

The expression of lncRNA TINCR in twenty cases of liver cancer tissues, matched liver cancer cell lines, and paracancerous tissues was analyzed by RT-PCR. CCK-8, clonogenic test, flow cytometry, and Transwell assay were used to measure the effect of lncRNA TINCR overexpression and knockdown on cell proliferation, apoptosis, and invasion. Luciferase reporter and Western blotting showed that lncRNA TINCR regulates the expression of ATG7 through miR-375, and the rescue experiment proved that lncRNA TINCR controls the invasion and proliferation of liver cancer cells via the miR-375/ATG7 signaling pathway. Furthermore, in vivo nude mouse assay demonstrated that overexpression of lncRNA TINCR inhibited liver cancer cell growth.

**Results:**

The lncRNA TINCR was highly expressed in liver cancer tissues and cell lines. Liver cancer cells responded differently to knockdown of the lncRNA TINCR compared to overexpression in terms of proliferation, colony formation, and invasion. miR-375 negatively affected the expression of ATG7. The lncRNA TINCR bound to miR-375 and influenced its expression. Transfection of miR-375 mimics greatly inhibited the inhibitory effect of lncRNA TINCR knockdown on the invasion and proliferation, whereas transfection of miR-375 inhibitor considerably reverses this effect on liver cancer cells. Overexpressing lncRNA TINCR increased liver cancer cell proliferation in vivo.

**Conclusion:**

By controlling the miR-375/ATG7 axis, the lncRNA TINCR impacts the proliferation and invasion of liver cancer cells. Therefore, the lncRNA TINCR/miR-375/ATG7 signaling axis could be a novel biological target for the diagnosis and therapy of liver cancer.

## 1. Introduction

Liver cancer is one of the most prevalent malignant tumors. There is an increasing incidence of liver cancer. About 90% of all primary liver tumors are hepatocellular carcinoma (HCC) [1, 2]. Numerous clinical studies have shown that liver cancer is more common in men than in women, and the disease has significant geographic difference, with the vast majority of cases (85%) occurring in East Asian and sub-Saharan African countries while Australia, Northern Europe, and the United States have a relatively low incidence [2]. Due to its stealthy development and lack of evident symptoms in the early stage, most patients identified with liver cancer have already advanced to the medium and late stage, missing the optimal period for surgical therapy [3, 4]. Furthermore, the surgical recurrence rate of patients with liver cancer reaches 70%, and the 5-year survival rate is less than 50% [5, 6]. Failure of therapy for liver cancer is often due to tumor recurrence and metastasis. Thus, increasing the early detection and therapeutic intervention of liver cancer requires studying novel indicators associated with the incidence and progression of liver cancer and discovering new intervention targets. Moreover, the treatment and prevention of liver cancer are critically dependent on increasing the patient's quality of life and duration of survival.

RNAs such as rRNA, tRNA, snRNA, snoRNA, microRNA, and long noncoding RNA (lncRNA) are all examples of noncoding RNA (ncRNA) [7–9]. It has been shown that lncRNAs may function as early disease diagnostic indicators and have roles in epigenetic, gene transcription, and posttranscriptional control of cells through several mechanism [10]. Many lncRNAs have been shown to be associated with malignant cellular proliferation, invasion, metastasis, angiogenesis, metabolism, and recurrence [7, 11, 12]. In liver cancer research, it has been reported that there are differentially expressed lncRNA profiles in liver cancer and adjacent tissues. Highly upregulated in liver cancer (HULC) may affect liver cancer cell proliferation and invasion by regulating downstream target genes [13–17]. This shows that HULC affects liver cancer in several ways. However, studies on lncRNAs in liver cancer are merely emerging. The etiology of liver cancer might be found in the discovery and identification of new lncRNAs with crucial activities and their distinct regulatory mechanisms.

Human-developed somatic cells produce a 3.7 kb lncRNA transcript termed terminal differentiation-induced noncoding RNA (TINCR), which binds to several miRNAs through a conserved region [18, 19]. The lncRNA TINCR plays a role in the stability of many miRNAs by binding to Stau1 protein [20]. In addition, as a competitive endogenous RNA in gastric cancer, the lncRNA TINCR has been demonstrated to influence the expression of PDK1 through miR-375, hence influencing gastric cancer cell proliferation and invasion [21]. miR-375 has a regulatory function in a number of cellular pathways [22–24]. Previous studies showed that miR-375 controls the expression of many different types of essential genes. Pathological alterations are often linked to abnormal miR-375 expression, making this miRNA a potential therapeutic target and biomarker [24]. For instance, the progression of HBV infection and HBV-related hepatocellular carcinoma may be reflected in the serum miR-375 level [25]. The lncRNA TINCR has been found in liver cancer tissues, although its role and molecular mechanism are unknown. Thus, the goals of this research are to (1) investigate lncRNA TINCR expression and biological activity in liver cancer tissue and (2) develop compounds for the early detection and targeted treatment of liver cancer target.

## 2. Material and Method

### 2.1. Clinical Specimen Collection

From June 2021 through January 2022, tumor and adjacent normal liver tissues from patients with liver cancer were collected from the hepatobiliary ward in the General Surgery Department of the First People's Hospital affiliated to Huzhou Normal College. All 20 cases included in the study were diagnosed as hepatocellular carcinoma by the pathology department of the hospital. There were no cancer cells in the paired adjacent tissue, and the distance from the edge of the cancer tissue was more than 2.0 cm. The selected cases had not received radiotherapy, chemotherapy, or other intervention before surgery. The hospital's ethics board approved the use of patient specimens after receiving written informed consents of patients. After isolation, samples were frozen in liquid nitrogen and stored in a -80°C freezer.

### 2.2. Cell Culture and Transfection

Liver cancer cell lines, including Hep2, Hep3B, SMMC-7721, Hub7, and H-97, and normal hepatocyte L02 were regularly cultured at 37°C in a 5% CO_2_ constant temperature cell incubator in DMEM (ScienCell, Carlsbad, CA, USA) complete media containing 10% FBS (Gibco, Carlsbad, CA, USA). A fresh medium was used every two days. After 90 percent confluence was reached at around 3–4 days, the cells were passaged using 0.25 percent trypsin.

After seeding logarithmically growing liver cancer cells (2 × 10^5^ cells/mL) in 6-well plates, the cells were transfected at 70% confluence and maintained in a 37°C, 5% CO_2_ incubator. After 6 h in the incubator, the media was changed to full cell culture medium and incubated for another 48 h.

### 2.3. Real-Time Quantitative PCR

When the lncRNA TINCR was expressed, it was identified using the ABI PRISM 7700 system with the SYBR-Green PCR Master Mix Kit after total RNA was extracted from tissues or cells following the protocol given by the TRIzol kit (Invitrogen, USA). The parameters include 45 cycles of amplification at 95°C for 5 minutes, 10 s at 60°C, and 30 s at 72°C. The lncRNAs TINCR and ATG7 were evaluated using GAPDH as an internal reference [17, 19], whereas U6 was used as a control of miR-375. Expression levels of lncRNA TINCR, miR-375, and ATG7 were determined using the 2^-△△Ct^ technique.

### 2.4. CCK-8 Detection of Cell Viability

Transfected liver cancer cells were plated at a density of 2 × 10^3^ cells/well in 96-well plates. Each of the 12 groups utilized three sets of wells for their culture, which lasted anywhere from 12 to 48 hours. CCK-8 reagent (Beyotime, Beijing, China) (10 *μ*L) was added after 2 hours of incubation, and the OD value was measured at 490 nm using a microplate reader. There were three separate iterations of the experiment, and the average of the results were recorded.

### 2.5. Clonogenic Experiments

Medium was replaced every three days after seeding 600 transfected liver cancer cells into each well of a 6-well plate. After 14 days in culture, the cells were fixed with 1 mL of 4% paraformaldehyde for 30 minutes and then washed three times with PBS. Crystal violet staining solution (Beyotime, Beijing, China) (1% in 1 mL) was used for staining for 10 minutes. The colonies were washed with PBS three times and counted.

### 2.6. Transwell Assay

A homogeneous coating of frozen Matrigel (BD Biosciences, Bedford, MA), diluted 1 : 8 with serum-free DMEM media, was applied to the Transwell chamber's microporous membrane and then incubated for 2 hours at 37°C. A total of 1 × 10^4^ cells/mL of serum-free medium was used to suspend the cells from each group; 100 *μ*L of each group's cell suspension was used to inoculate the top chamber of the Transwell chamber (8 *μ*m pore sizes, Corning, New York, USA), while 700 *μ*L of full cell culture medium was added to the bottom chamber. Cells were incubated for 48 hours. After removing the medium, cotton swabs were used to carefully wipe off the microporous membrane's outermost layer of cells. The cells were fixed with 95% ethanol after being rinsed twice in PBS. The cells were then washed in phosphate-buffered saline (PBS), stained with 1% crystal violet staining solution (Beyotime, Beijing, China), and examined using an inverted microscope.

### 2.7. RNA-FISH

The localization of lncRNA TINCR in liver cancer cells was detected by immunofluorescence. Cy3 (red) was used to label lncRNA TINCR, and DAPI (blue) was used to display the nucleus, which was observed under a fluorescence microscope.

### 2.8. Western Blot

Total protein was extracted using RIPA reagent (Beyotime, Shanghai, China). The protein concentration was determined using the BCA test, and a 10% SDS-PAGE electrophoresis gel was prepared. After an overnight incubation with primary antibodies anti-ATG7 (CST, 1 : 800) and anti-GAPDH (CST, 1 : 1000) at 4°C, wash the membrane and let it settle in a shaker at 37°C with a dilution of horseradish peroxidase-labeled secondary antibody IgG (1 : 2000) for 1 hour. After the addition of ECL reagent, the membrane was developed and imaged using a gel imaging equipment; the expression of ATG7 in comparison to GAPDH was evaluated using ImageJ.

### 2.9. Dual-Luciferase Reporter Gene Assay

The lncRNA TINCR was predicted to have a continuous binding site with the miR-375 nucleotide sequence using LncBase v.2. A continuous binding site with miR-375 was predicted to exist in the 3′ untranslated region (3′ UTR) of ATG7 using the bioinformatics tool TarBase v.8. Cotransfection of HepG2 cells with lncRNA TINCR-WT, lncRNA TINCR-Mut, ATG7-WT, ATG-MUT, and miR-375 mimic was performed using the Lipofectamine™ 2000 kit as per the manufacturer's instructions (Invitrogen, USA). The transfection medium was changed to fresh media 12 hours later. Cells were extracted after 48 hours. The luciferase activity was detected following the manufacturer's instructions (Promega, USA).

### 2.10. In Vivo Nude Mouse Experiment

The 8-12-week-old NOD/SCID mice were used, and TINCR-overexpressing liver cancer cells and their control cells (1 × 10^6^) were injected subcutaneously. The tumor volume was taken using the formula *V* = 1/2 × *a* × *b*2, where *a* is the long diameter and *b* is the short diameter. Tumor growth curves were created using the available data. Mice were euthanized after 4 weeks, and their tumors were weighed. After that, mRNA was isolated from the samples, and RT-PCR was used to examine miR-375 and ATG7 levels in the samples.

### 2.11. Statistical Analysis

The SPSS 20.0 software was used for analysis. The data was presented as mean ± standard deviation (*x* ± *s*). Groups were compared using one-way analysis of variance, and statistical differences in measurement were analyzed using the *t*-test; differences at the *P* < 0.05 level were considered significant.

## 3. Results

### 3.1. TINCR Expression in Cell Lines and Liver Cancer Tissues

**Figure 1:**
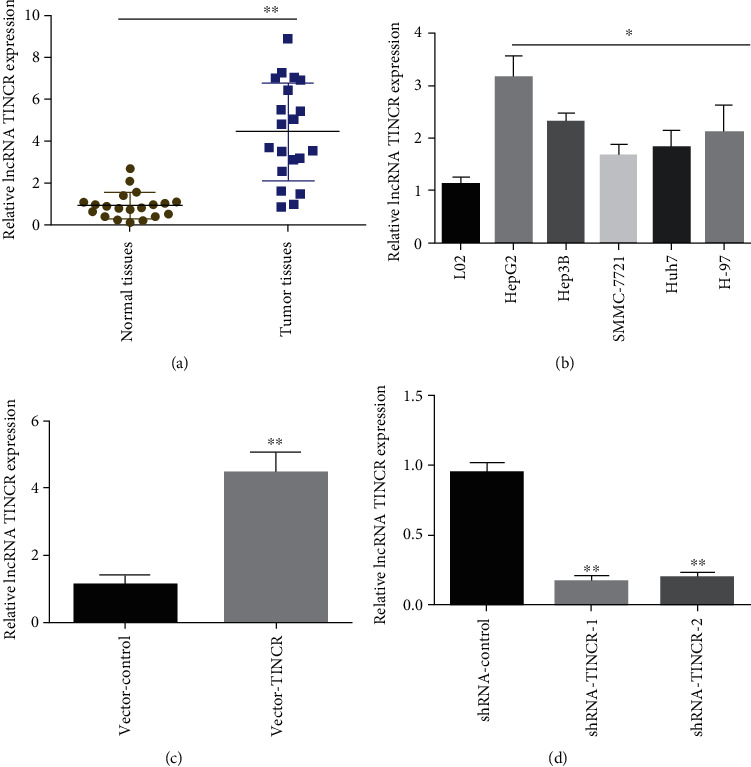
lncRNA TINCR is highly expressed in liver cancer tissues and cell lines. (a) lncRNA TINCR expression levels in liver cancer tissues and adjacent tissues detected using RT-PCR. (b) Relative lncRNA TINCR expression in cancerous liver cells and normal liver L02 cells. (c) The expression of lncRNA TINCR in liver cancer SMMC-7721 cells after transfection with lentiviruses overexpressing lncRNA TINCR. (d) The expression of lncRNA in HepG2 liver cancer cells transfected with shRNA-TINCR-1 and shRNA-TINCR-2; *n* = 3, ^∗^*P* < 0.05, and ^∗∗^*P* < 0.01.

In the first step, RNA was isolated from 20 liver cancer samples and paracancerous tissue samples. RT-PCR showed that malignant tissues overexpressed lncRNA TINCR compared to paracancerous normal tissues (Figure 1(a)). Using RT-PCR, TINCR expression was evaluated in liver cancer cell lines (Hep2, Hep3B, SMMC-7721, Hub7, and H-97) and normal liver cells L02. SMMC-7721 liver cancer cells had the lowest, whereas HepG2 cells had the greatest TINCR expression (Figure 1(b)). A lentiviral vector was developed for the overexpression of lncRNA TINCR, which was then transfected into SMMC-7721 cells from patients with liver cancer. Meanwhile, a lentiviral vector targeting TINCR was created and transfected into HepG2 cells from liver cancer patients. Then, the amount of TINCR in the system was determined by using RT-PCR. Compared to transfection with a blank control vector (vector-control), lncRNA TINCR expression was significantly upregulated in SMMC-7721 cells after transfection with an lncRNA TINCR (vector-TINCR) vector (Figure 1(c)). Compared to the control group, the expression level of lncRNA TINCR was significantly decreased in HepG2 cells after transfection with lentiviral vectors targeting TINCR (shRNA-TINCR-1 and shRNA-TINCR-2) (Figure 1(d)). These findings demonstrate that lncRNA TINCR is highly expressed in HCC tumor tissues and cell lines.

### 3.2. lncRNA TINCR Promotes Hepatocellular Cancer Cell Growth and Invasion

**Figure 2:**
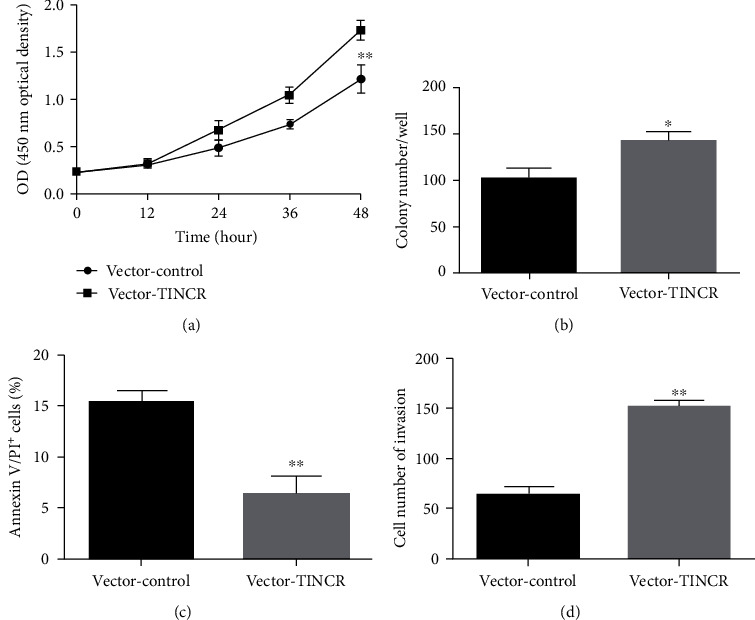
Knockdown lncRNA TINCR inhibits the proliferation of liver cancer SMMC-7721 cells in vitro. (a) The cell viability was measured using CCK-8 assay. (b) Colony-formation assay was used to measure cell proliferation. (c) The apoptosis was measured using flow cytometry. (d) Transwell was used to assess the impact of lncRNA TINCR liver cancer cell invasion; *n* = 3, ^∗^*P* < 0.05, and ^∗∗^*P* < 0.01.

To further examine the impact of lncRNA TINCR overexpression on the proliferation and invasion of liver cancer SMMC-7721 cells, we used CCK-8 to measure cell proliferation caused by lncRNA TINCR overexpression. Liver cancer cell proliferation was shown to be significantly elevated when lncRNA TINCR was overexpressed compared to the vector-control group (Figure 2(a)). Overexpression of lncRNA TINCR resulted in significantly more clones of liver cancer cells forming compared to the vector-control group in a clonogenic assay (Figure 2(b)). Overexpression of lncRNA TINCR induced apoptosis in SMMC-7721 cells, a liver cancer cell line, while it significantly suppressed apoptosis of liver cancer cells, as compared to the vector-control group (Figure 2(c)). Transwell was used to determine the impact of lncRNA TINCR on the invasion capacity of liver cancer cells, and the findings indicated that lncRNA TINCR overexpression increased the invasion ability of liver cancer cells in comparison to the vector-control group (Figure 2(d)). The above data show that liver cancer cell proliferation, invasion, and survival may be considerably boosted by overexpressing lncRNA TINCR while apoptosis is suppressed.

### 3.3. lncRNA Knockdown TINCR Inhibits Hepatocellular Cancer Growth and Invasion

**Figure 3:**
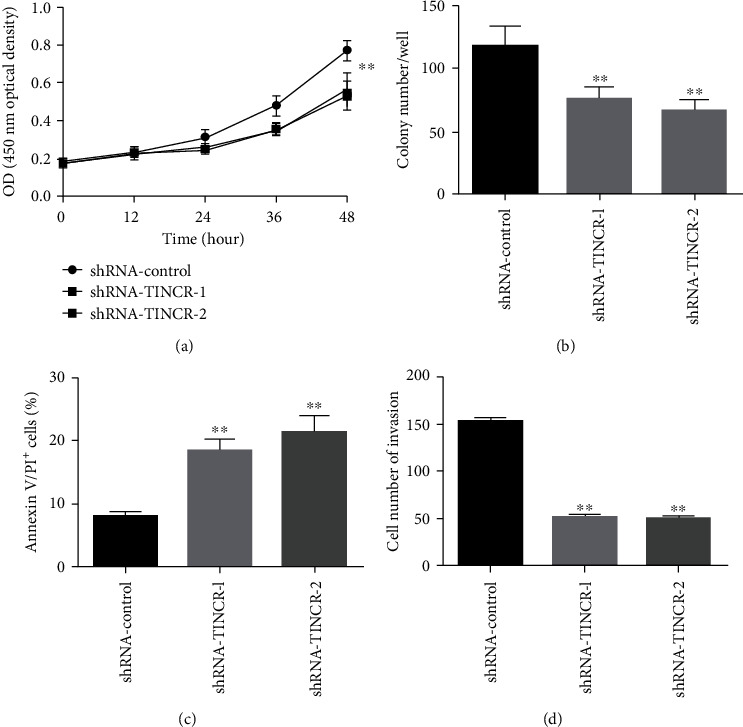
Overexpression of lncRNA TINCR promotes the proliferation of liver cancer Hep G2 cells in vitro. (a) The cell viability measured using CCK-8 assay. (b) Colony-formation assay used to measure cell proliferation. (c) The apoptosis measured using flow cytometry. (d) Transwell assay assessed the impact of lncRNA TINCR on liver cancer cell invasion; *n* = 3, ^∗^*P* < 0.05, and ^∗∗^*P* < 0.01.

The impact of knockdown of lncRNA TINCR on the proliferation capacity of liver cancer cells was identified by CCK-8 in order to better investigate the ability of knockdown of lncRNA TINCR on the proliferation and invasion of liver cancer HepG2 cell. The findings demonstrated that liver cancer cell proliferation was dramatically suppressed when lncRNA TINCR was knocked down (shRNA-TINCR-1 and shRNA-TINCR-2) (Figure 3(a)). Knockdown of lncRNA TINCR substantially suppressed colony formation in liver cancer cells compared to the blank control group (shRNA-control) as shown by the clonogenic test (Figure 3(b)). Flow cytometry showed that silencing the lncRNA TINCR caused liver cancer cells to self-destruct. We found that knocking down lncRNA TINCR (shRNA-TINCR-1 and shRNA-TINCR-2) increased significantly liver cancer cell apoptosis as compared to the control group (shRNA-control) (Figure 3(c)). Transwell assays showed that knocking down lncRNA TINCR reduced the capacity of liver cancer cells to invade. It was found that knocking down lncRNA TINCR (shRNA-TINCR-1 and shRNA-TINCR-2) reduced the invasive capacity of cells compared to the control group (Figure 3(d)). These results suggested that liver cancer cell growth, invasion, and apoptosis were suppressed by targeting the lncRNA TINCR.

### 3.4. The lncRNA TINCR Regulates miR-375 in Hepatocellular Cancer Cells

**Figure 4:**
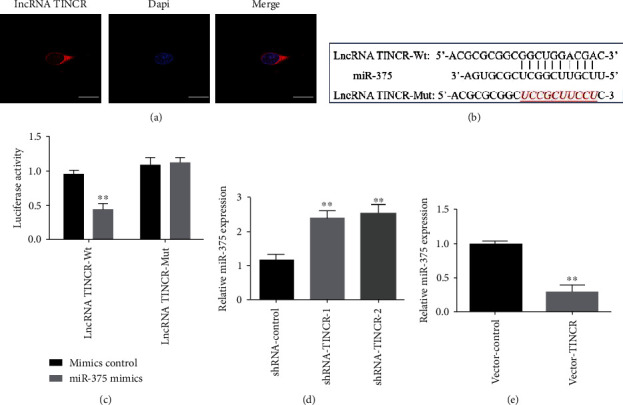
lncRNA TINCR localized in the cytoplasm and interacted with miR-375. (a) Fluorescence in situ hybridization (FISH) identified the location of lncRNA-TINCR in liver cancer HepG2 cells. Blue represents the nucleus, and red is lncRNA-TINCR. (b) The potential binding region with miR-375 and lncRNA TINCR and the mutant form of lncRNA TINCR were shown. (c) Luciferase reporter gene test for determining miR-375's ability to bind to lncRNA TINCR. (d) Knockdown lncRNA TINCR affects the expression of miR-375. (e) Effect of overexpressing the lncRNA TINCR on the expression of miR-375. Scale bar = 50 *μ*m; *n* = 3, ^∗^*P* < 0.05, and ^∗∗^*P* < 0.01.

To further understand the molecular mechanism by which lncRNA TINCR plays a role in liver cancer cells, we used RNA-FISH to map its distribution inside the tumor cells. It was found that lncRNA TINCR was mostly localize in cytoplasm (Figure 4(a)). lncRNA may function as a ceRNA to control the production of miRNAs. The lncRNA TINCR was discovered to have a binding site with miR-375; thus, we created a luciferase reporter gene vector and mutation site according to bioinformatics predictions (Figure 4(b)). Compared to the control group, the addition of miR-375 mimics significantly downregulated intracellular luciferase activity in cells harboring the lncRNA TINCR wild type (lncRNA TINCR-Wt). The lncRNA TINCR-Mut group showed no significant change in intracellular luciferase activity in response to the addition of miR-375 mimics (Figure 4(c)). Through further RT-PCR analysis, we found that knockdown of lncRNA TINCR (shRNA-TINCR-1 and shRNA-TINCR-2) could significantly increase the expression of miR-375 in cells compared with the shRNA-control group (Figure 4(d)), whereas overexpression of lncRNA TINCR (vector-TINCR) significantly decreased the expression of intracellular miR-375 (Figure 4(e)). These results indicate that lncRNA TINCR has a role in regulating miR-375 expression in liver cancer cells.

### 3.5. miR-375 Regulates the Expression of ATG7 in Hepatocellular Carcinoma Cells

**Figure 5:**
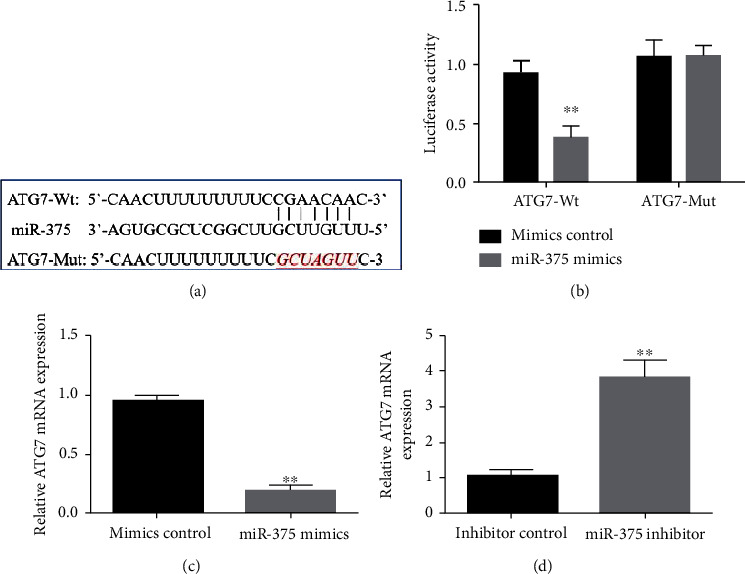
miR-375 controls the expression of ATG7 in liver cancer cells. (a) The binding sites between miR-375 and ATG7 obtained from starBase database. (b) The binding activity of miR-375 and ATG7 measured by the luciferase reporter experiment. (c, d) Effects of adding miR-375 mimics or inhibitors on intracellular ATG7 expression; *n* = 3, ^∗^*P* < 0.05, and ^∗∗^*P* < 0.01.

miRNAs have been demonstrated to work not by encoding proteins but by controlling the production of mRNAs in downstream target genes. Therefore, bioinformatics algorithms indicated that miR-375 may bind to the downstream target gene ATG7 in liver cancer cells (Figure 5(a)). When miR-375 mimics were added to cells from the ATG7 mutant (ATG7-Mut) group, the effect was to significantly reduce luciferase activity inside the cells, as compared to the control group. Intracellular luciferase activity was not significantly altered by miR-375 mimics (Figure 5(b)). Intracellular ATG7 expression, as measured by RT-PCR, was significantly downregulated by miR-375 mimics (mimics) compared to the mimics-control group (Figure 5(c)), whereas it was markedly upregulated by miR-375 inhibitors (Figure 5(d)). These findings suggest that miR-375 can control ATG7 expression in liver cancer cells.

### 3.6. The lncRNA TINCR Regulates the Expression of ATG7 via miR-375 in Hepatocellular Carcinoma Cells

**Figure 6:**
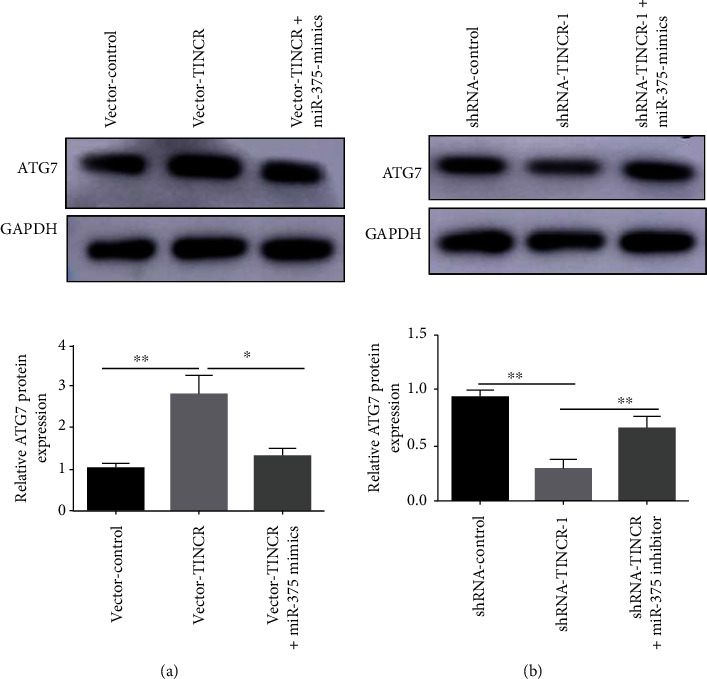
lncRNA TINCR functions as a ceRNA through sponging miR-375 to regulate ATG7 in liver cancer cells. (a) Western blot assay detecting the expression of ATG7 in SMMC-7721 cells after transfected with miR-375 mimics or overexpressing lncRNA TINCR (vector-TINCR). (b) Western blot assay detecting the expression of ATG7 after transfected with miR-375 inhibitor or knockdown lncRNA TINCR (shRNA-TINCR-1) in HepG2 cells; *n* = 3, ^∗^*P* < 0.05, and ^∗∗^*P* < 0.01.

Western blotting was used to investigate the role of miR-375 in the regulation of ATG7 expression by lncRNA TINCR in liver cancer cells. The results indicated that overexpressing lncRNA TINCR (vector-TINCR) in liver cancer SMMC-7721 cells significantly increased ATG7 expression compared to the vector control group (vector-control) (Figure 6(a)). Compared with the shRNA-control group, suppression of lncRNA TINCR (shRNA-TINCR-1) reduced ATG7 expression in HepG2 cells. However, the inhibitory impact of lncRNA TINCR (shRNA-TINCR-1) knockdown on ATG7 expression was markedly reversed by adding miR-375 inhibitor to the knockdown lncRNA TINCR (shRNA-TINCR-1) group (Figure 6(b)). These findings indicated that lncRNA TINCR controls ATG7 expression in liver cancer cells via targeting miR-375.

### 3.7. lncRNA TINCR Regulates the Proliferation and Invasion of Liver Cancer Cells through miR-375/ATG7

**Figure 7:**
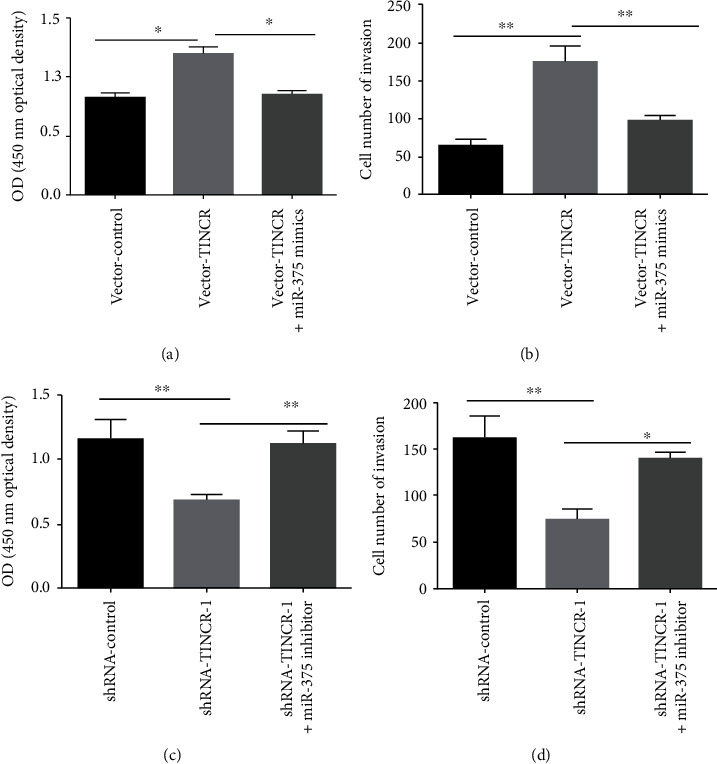
lncRNA TINCR affects liver cancer cell proliferation and invasion via targeting miR-375/ATG7. (a, b) miR-375 mimics significantly blocked the promotion effect on proliferation and invasion in lncRNA TINCR overexpressing SMMC-7721 cells (vector-TINCR). (c, d) miR-375 inhibitor significantly blocked the inhibition on proliferation and invasion in shRNA-TINCR-1 knockdown cells; *n* = 3, ^∗^*P* < 0.05, and ^∗∗^*P* < 0.01.

We investigate the role of lncRNA TINCR in miR-375/ATG7-mediated regulation of hepatocellular carcinoma proliferation and invasion. Overexpression of lncRNA TINCR (vector-TINCR) promoted cell proliferation and invasion in SMMC-7721 cells compared to vector-control. Overexpression of the lncRNA TINCR (vector-TINCR) promotes cell proliferation and invasion; however, adding miR-375 mimics to the TINCR (vector-TINCR) group greatly inhibits these effects (Figures 7(a) and 7(b)). TINCR knockdown (shRNA-TINCR-1) HepG2 liver cancer cells proliferated and invaded less than the control group (shRNA-control). miR-375 inhibitors effectively reverse the suppressive impact of lncRNA TINCR (shRNA-TINCR-1) knockdown on cell proliferation and invasion (Figures 7(c) and 7(d)). These results suggest that hepatocellular carcinoma lncRNA TINCR controls cell proliferation and invasion through miR-375/ATG7.

### 3.8. TINCR Controls Liver Cancer Cell Proliferation through miR-375/ATG7

**Figure 8:**
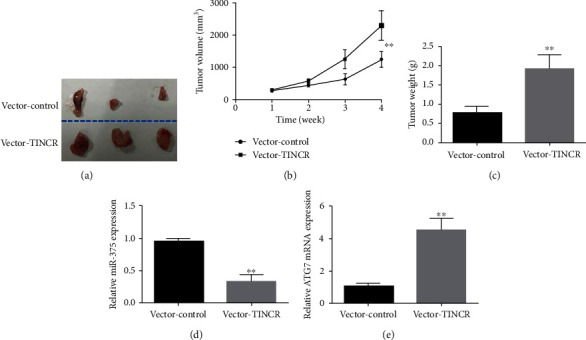
The lncRNA TINCR regulates the proliferation of liver cancer cells *in vivo* through miR-375/ATG7. (a) The size of tumor tissue after injecting lncRNA TINCR-overexpressing liver cancer SMMC-7721 cells and its control cells into the abdomen of NOD/SCID mice for 4 weeks, (b) the growth curve of the tumor tissue, (c) tumor weight, (d) the expression of miR-375 in tumor tissue, and (e) the expression of AGT7 in tumor tissue; *n* = 3, ^∗^*P* < 0.05, and ^∗∗^*P* < 0.01.

Subcutaneous injections of SMMC-7721 cells (a liver cancer cell line that overexpresses lncRNA TINCR) or control cells (without expressing TINCR) were made into NOD/SCID mice. Tumor size and weight were measured weekly, and the mice's survival rates were tracked. Overexpression of lncRNA TINCR was shown to significantly increase liver cancer cell proliferation in vivo, in comparison to the control group (Figures 8(a) and 8(b)). The tissue containing the tumor was removed and weighed. Overexpression of lncRNA TINCR increased tumor weight relative to the blank control group (vector-control) (Figure 8(c)). Further RT-PCR showed a substantial decrease in miR-375 expression in tumor-bearing tissues of the lncRNA TINCR overexpression group compared to the vector-control group (Figure 8(d)), whereas ATG7 expression was shown to be considerably upregulated in tumor tissues that overexpressed lncRNA TINCR (vector-TINCR) (Figure 8(e)). These results demonstrated that lncRNA TINCR controlled liver cancer cell proliferation through miR-375/ATG7 in vivo.

## 4. Discussion

The prognosis of patients diagnosed with hepatocellular carcinoma is dismal. A novel approach to managing hepatocellular carcinoma in its intermediate and advanced stages is targeted therapy. Finding more precise liver cancer targets for clinical targeted treatment of liver cancer is important. Multiple studies have linked lncRNAs to liver cancer, its progression, metastasis, and apoptosis [26]. According to the results of this investigation, lncRNA TINCR is substantially expressed in liver cancer tissues and cells. When this gene is overexpressed, it inhibits apoptosis and promotes cell proliferation, clonal expansion, and invasion. The cytoplasmic lncRNA TINCR functions as a ceRNA to suppress miR-375 expression. Studies have shown that miR-375 may control the expression of ATG7 in a particular manner. Experiments on cell function showed that lncRNA TINCR regulates miR-375/ATG7, which in turn influences liver cancer cell proliferation and invasion. Overexpression of lncRNA TINCR significantly stimulated liver cancer cell proliferation in vivo.

The recently discovered lncRNA TINCR has a crucial role in the initiation and progression of a wide variety of cancers [27]. For instance, the lncRNA TINCR may operate as a competitive endogenous RNA in human breast cancer by recruiting DNMT1 and driving carcinogenesis through the STAT3-TINCR-EGFR signaling axis [28]. Additionally, lncRNA TINCR may be employed as a tumor marker for preclinical evaluation [19]. Multivariate study indicated that serum lncRNA TINCR predicts breast cancer survival [29]. Increased serum lncRNA TINCR levels were associated with poor patient clinicopathological characteristics and clinical outcomes. Patients with lymph node involvement, advanced clinical T stage, distant metastases, poor prognosis, and a high Gleason score are likely to have high levels of the lncRNA TINCR. Furthermore, tissue expression levels of TRIP13 mRNA and protein were inversely linked with lncRNA TINCR expression levels. lncRNA TINCR suppresses the transcription and translation of TRIP13 in prostate cancer cells [30]. Cell function tests show that overexpression of lncRNA TINCR greatly boosted the proliferation, colony formation, invasion, and inhibition of apoptosis of liver cancer cells, whereas knockdown of lncRNA TINCR had the opposite effect.

Previous investigations revealed that lncRNAs are aberrantly expressed in cancers, acting as tumor-promoting genes or tumor-suppressor genes to regulate cell behavior and promote cancer development by interacting with miRNAs and mRNAs [31]. For example, in bladder cancer, lncRNA TINCR promotes tumor proliferation and invasion by regulating miR-7 and mTOR [32]. In this study, the majority of TINCR lncRNA was found in the cytoplasm. Bioinformatics analyses suggested that lncRNA TINCR controls the levels of miR-375 in the body. miR-375 is involved in several processes, according to studies, including pancreatic islet formation, glucose homeostasis, mucosal immunity, pulmonary surfactant secretion, and carcinogenesis [24, 33]. Recent research showed that miR-375 is strongly downregulated in a wide range of cancers and that it suppresses carcinogenesis by targeting various critical oncogenes including AEG-1, YAP1, IGF1R, and PDK1 [22, 34]. Additional experiments in this work demonstrated that miR-375 controls the expression of ATG7. Functional experiments demonstrated that knocking down lncRNA TINCR significantly inhibited ATG7 expression in liver cancer cells, while transfecting miR-375 mimics significantly blocked this effect. In addition to regulating immunity, cell death, and protein secretion in collaboration with other ATG proteins, ATG7 also controls the cell cycle and apoptosis [35]. miR-375 mimics reduce the stimulatory impact of lncRNA TINCR on liver cancer cell proliferation and invasion, while transfection of a miR-375 inhibitor significantly reverts this effect. Overexpression of lncRNA TINCR greatly enhances liver cancer cell proliferation, as shown by tests in vivo using nude mice. In conclusion, TINCR reduces miR-375 expression in liver cancer cells, which lowers ATG7 to limit cell proliferation and invasion.

In conclusion, this research showed that lncRNA TINCR was highly expressed in hepatocellular cell lines and cancer tissues. Overexpression or silencing of lncRNA TINCR strongly influenced cell proliferation, colony formation, apoptosis, and invasion. The molecular mechanism was that lncRNA TINCR negatively influenced miR-375 expression and regulated ATG7 expression. Overall, our work provided evidence that the lncRNA TINCR controls the growth and invasion of liver cancer cells through the miR-375/ATG7 signaling pathway. In context of this, lncRNA TINCR shows promise as a novel target for precision therapy of liver cancer.

## Data Availability

All experimental data used to support the findings of this study are available from the corresponding author upon request.
